# University Matriculation

**Published:** 1902-03-01

**Authors:** 


### University Matriculation.

The announcement said to have been made by the Medical Faculty of the new London University, to the effect that the most serious impediment in the way of what may be called the general graduation of the medical profession may be traced to a complete absence of correspondence between the ordinary teaching of a fair middle-class school and the ordinary requirements of a matriculation examination, will not come as a surprise to any who are practically familiar with the general character of the mental training, or of what passes for it, which has been received by a very large proportion of the young men who present themselves to the teachers of the various London and provincial schools with the coming of each recurrent October. These young men are frequently quite unfit to encounter the ordeal to which, for the present at least, it may be presumed that any University would subject them; and it would not always be easy to say whether the larger number of instances of such unfitness were traceable to incapacity to cover the ground required for the examination, or to such deficiency of training as would prevent them from approaching any one of the subjects in a satisfactory and workmanlike manner; whether, in short, the candidates had not been taught a sufficient number of things, or had never been taught the way to learn. As a rule, probably, the middle class schoolmaster aims at imparting a smattering of everything which can be required, and imparts it in such a fashion that the period of a vacation is sufficient to permit the boy to forget everything that he is supposed to have learned. The blatant and more or less illiterate grievance-mongers of the profession, who go about to meetings, and move and carry foolish and futile resolutions, are never tired of demanding that the title conferred by a degree should be rendered universal; but they have not yet, so far as we have seen, disclosed any patent method of reconciling universal graduation with a prevailing want of knowledge of the subjects in which graduates must be assumed to be proficient. It may be, with the constantly increasing number of provincial universities, that the standards of some of them will become relaxed under the influence of competition; and that the state of things which has been brought about, as far as the essentials of medicine and surgery are concerned, by the rivalry of 19 examining and licensing bodies, may before long repeat itself in relation to *literæ humaniores*, or to those preliminary studies which have been shown, by the experience of many ages, to prepare a soil in which other kinds of knowledge may be planted with some reasonable hope of a successful harvest.

It has been impossible to read between the lines of recent discussions in the General Medical Council without perceiving that the whole question of preliminary education for the medical profession is one of the very first importance, alike in its bearing upon the efficiency of "qualified practitioners" for their duties, and upon the position which they will be able to take and hold in relation to the more highly educated members of the society in which their lot will be cast. It is not difficult to see that some of the licensing bodies have but slender faith in the common assertion that the public will discover excellence if it be presented to them, and, having discovered, will pay for it at a price commensurate with its value. Not a single licensing body, as far as we are aware, has made any attempt to render its qualification or qualifications distinctly more difficult of attainment, and correspondingly more valuable when attained, than those of its rivals, unless this has been done with the definite intention of appealing only to a very small class of candidates, who are likely to seek for exceptional positions. There have been men in the Council, and probably there are some still remaining, who have been able to see that the question of preliminary medical education is really the most important of its kind which can be raised, and that the future status of the profession must be almost wholly governed by the manner in which that question is dealt with by those in whose hands it mainly rests. If the existing condition of things is to be maintained, and if imperfectly educated young men of humble parentage, strongly influenced by the commercial instincts which they have inherited from their forefathers, are to be permitted or encouraged to pass through a net the meshes of which have been made wide for their accommodation, the inevitable result will be a reproduction of conditions which may be studied with advantage on many parts of the Continent, and which are not wholly unknown even amongst ourselves. It is impossible to cast a glance at some of the forms of practice which obtain even now in the metropolis, to think of the mushroom "specialists" and "specialisms" which are every day more and more conspicuous, to consider many prevailing methods of operative treatment by the light either of science or of common sense, to read the little books which flow in unceasing stream from the press, and to give occasional ear to the narratives of patients, without arriving at a conviction that many things in relation to the profession are moving upon a downward grade, and that those who occupy its highest places are far too indulgent to the pretensions of sham science, or to the practices of that commercial morality which seems to be undermining the roots of professional honour. "It is not enough" once said Prescott Hewett, "for a surgeon to be honest, he must be chivalrous." It is the most piteous sight of the twentieth century to watch those who should be the Bayards of their calling, and to see them scrambling in the gutter for such rewards as will naturally satisfy the desires of the *Kaufleute*.

